# Potentially Toxic Elements in Urban-Grown Lettuce: Effectiveness of Washing Procedures, Risk Assessment, and Isotopic Fingerprint

**DOI:** 10.3390/plants13192807

**Published:** 2024-10-07

**Authors:** Camila Neves Lange, Bruna Moreira Freire, Lucilena Rebelo Monteiro, Marycel Elena Barboza Cotrim, Bruno Lemos Batista

**Affiliations:** 1 Center for Natural and Human Sciences (CCNH), Federal University of ABC (UFABC), Santo André 09210-580, Brazil; bruna.freire@ufabc.edu.br; 2 Instituto de Pesquisas Energéticas e Nucleares IPEN-CNEN, São Paulo 05508-000, Brazil; lrmonteiro@ipen.br (L.R.M.); mecotrim@ipen.br (M.E.B.C.)

**Keywords:** heavy metal contamination, urban garden produce, health risk assessment, contaminant removal techniques, isotopic tracing

## Abstract

This study investigates the presence of potentially toxic elements (PTEs) in lettuce (*Lactuca sativa* L.) grown in urban gardens in a highly industrialized city in Brazil and evaluates the effectiveness of different washing methods in reducing contamination. Ten elements (arsenic (As), barium (Ba), cadmium (Cd), cobalt (Co), chromium (Cr), copper (Cu), nickel (Ni), lead (Pb), vanadium (V), and zinc (Zn)) were analyzed for their concentration, and a health risk assessment was performed. The results showed that Pb concentrations in lettuce from gardens near the Capuava Petrochemical Complex reached 0.77 mg kg^−1^, exceeding both national and international safety limits. The most effective washing procedure involved the use of sodium hypochlorite, which reduced As by 46%, Pb by 48%, and V by 52%. However, elements such as Ba, Cd, Cr, and Ni showed limited reductions of less than 10% across all washing methods. Health risk assessments revealed a particular concern for children, with the total cancer risk (TCR) exceeding acceptable limits in some gardens. Isotopic analysis of Pb revealed that atmospheric pollution from gasoline emissions and industrial activities were the primary sources of contamination. The elevated levels of Pb, Cr, and As highlight the need for targeted health education in local communities, especially regarding the importance of proper washing techniques. Risk management strategies, including improved contamination control and public awareness, are crucial to minimize exposure to these harmful elements, particularly in vulnerable populations like children.

## 1. Introduction

Urban agriculture has emerged as a sustainable solution for addressing food insecurity in modern cities by providing local, fresh produce and reducing dependency on long supply chains [1,2,3]. It also offers environmental benefits such as mitigating climate change impacts by absorbing carbon and managing urban heat islands through green spaces [3]. Moreover, urban agriculture promotes improved nutrition and physical activity, fostering stronger community ties through shared green spaces, which can enhance social cohesion. These benefits contribute to the resilience of urban environments and their ability to sustainably meet the challenges of the future [4].

However, urban food production is not without challenges, particularly when it comes to the risk of contamination by potentially toxic elements (PTEs) [5], which can accumulate in urban soils due to industrial activities, heavy vehicular traffic, and other anthropogenic [6]. In Brazil, cities such as such as Belo Horizonte [7], Campinas [8], São Paulo [9,10], Santo André [11], and Recife [12], among others, highlight the pervasive impact of industrial activities, heavy vehicular traffic, and other anthropogenic sources. These pollutants pose risks to urban agriculture, public health, and environmental sustainability, requiring comprehensive monitoring and mitigation strategies.

The São Paulo Metropolitan Area (MASP), home to over 22 million people, is one of the largest urban regions in the world [13]. This region faces significant pollution pressures, with over 7 million vehicles contributing to airborne contamination. Studies conducted in the MASP show alarming levels of pollutants such as As and Pb during the winter months, reflecting the impact of both vehicular emissions and industrial activities [14]. The southeastern part of the MASP, where the “big ABC” region is located, including Santo André, is particularly vulnerable due to its proximity to the Capuava Petrochemical Complex, the largest industrial hub in the area [15,16]. This industrial complex plays a significant role in contaminating the environment and urban gardens with PTEs such as Pb, Cr, and As [11]. Therefore, there is a pressing need to monitor and manage the bioaccumulation of PTEs in urban horticulture to safeguard public health.

Lettuce (*Lactuca sativa* L.) is widely grown in Brazil due to its adaptability, low production costs, and resistance to pests [17]. However, lettuce is particularly susceptible to contamination from PTEs, either through direct contact with contaminated soil or through atmospheric deposition [18,19,20,21]. Studies have indicated that PTEs such as Pb, Cd, Cr, and As can accumulate in lettuce grown in urban environments, posing potential health risks to consumers, especially children [18,19,22]. These risks highlight the need to better understand how PTEs accumulate in urban-grown lettuce and how effective common mitigation strategies, such as washing, are at reducing contamination [18,19,22].

Isotopic analysis provides a powerful tool for tracing the sources of PTE contamination in urban environments and food crops [23,24], for improving understanding of biogeochemical processes in soil–plant systems [25], and for identifying organic versus conventional production methods [26]. By studying the isotopic ratios of elements such as Pb, it is possible to discriminate between different sources of contamination, including industrial emissions, historical use of leaded gasoline, and natural geological contributions [27,28]. The natural occurrence and the various activities associated with the use of Pb make it difficult to identify the source based solely on elemental content or association with other PTEs. Therefore, the isotopic fingerprinting is often based on the Pb stable isotopes. The origin of Pb stable isotopes can be considered as primordial (^204^Pb) or radiogenic from the decay of ^238^U, ^235^U, and ^232^Th, corresponding to ^206^Pb, ^207^Pb, and ^208^Pb, respectively [27,28,29,30].

This study investigates the accumulation of PTEs in lettuce from urban gardens near industrial areas, focusing on elements such as As, Ba, Cd, Co, Cr, Cu, Ni, Pb, V, and Zn. The goals are to assess contamination levels, evaluate health risks from ingestion, and test the effectiveness of different washing methods in reducing PTEs, particularly in gardens with high contamination. In addition, the study uses Pb isotopic analysis to identify sources of contamination, in order to improve understanding of the risk associated with urban agriculture and provide recommendations for minimizing exposure to harmful elements.

## 2. Results

### 2.1. Concentration of Trace Elements in Lettuce

In this study, PTE concentrations in lettuce leaves collected from urban gardens in Santo André, Brazil, were determined on a dry matter basis. Fresh samples were initially weighed and dried, with minimal variation in moisture content (90 ± 3 *w*/*w*%). The concentrations measured on a fresh mass basis were estimated to be approximately one-tenth of the dry mass values, so the dry mass values were divided by 10 to correspond to the consumable fresh mass (Table 1). In Brazil, the regulations for maximum allowable levels of inorganic contaminants in food are established by ANVISA’s RDC Resolution n° 42 of 2013 [31] and Decree Law 55871 of 1965 [32]. The Codex Alimentarius Commission establishes the internationally recognized maximum levels [33]. These limits apply to products as they are offered to consumers, which, in the case of vegetables such as lettuce, are consumed fresh.

The results showed significant variation in PTE concentrations among the different gardens. The highest concentrations of Pb, Cr, and As were detected in samples from Capuava 1, with Pb concentrations reaching 0.77 mg kg^−1^ (fresh mass). This level exceeds both national and international regulatory limits for Pb in vegetables (0.1 mg kg^−1^), indicating a serious contamination issue in this area. In comparison, other gardens (Capuava 2, Marajoara, Bairro Jardim, Vila Bastos, and Bela Vista) had significantly lower concentrations of Pb, with levels ranging between 0.009 and 0.053 mg kg^−1^, which are within the acceptable safety limits.

The elevated Pb levels in Capuava 1 suggest a localized source of contamination, likely linked to the nearby Capuava Petrochemical Complex, which is known for its industrial emissions [11]. Additionally, the presence of high Cr levels (0.07 mg kg^−1^) in Capuava 1 further supports this hypothesis, as Cr is commonly associated with industrial pollution [11]. The fact that As concentrations also exceeded recommended limits in some samples (0.009 mg kg^−1^) further underscores the significant pollution burden in this particular area.

In contrast, the lower PTE concentrations in the other gardens suggest that contamination is not uniformly distributed across the region. For example, the gardens in Bairro Jardim and Marajoara, which are located further from industrial activities, showed considerably lower contamination levels. Pb levels in these areas were well below regulatory thresholds (0.022 mg kg^−1^ and 0.009 mg kg^−1^, respectively), highlighting the role of proximity to pollution sources as a major determinant of contamination risk.

While Pb levels in most gardens were under regulatory limits, the elevated levels in Capuava 1 are concerning, particularly for vulnerable populations like children, who are more susceptible to the harmful effects of PTE exposure. Pb, in particular, is a neurotoxin, and even low levels of exposure can lead to significant developmental and cognitive impairments in children [34]. These findings suggest a pressing need for targeted monitoring and risk mitigation strategies in areas near industrial sources.

In addition to Pb, Cd concentrations also varied significantly across the gardens, with the highest levels found in Capuava 1 (0.062 mg kg^−1^), which, although below international limits (0.1 mg kg^−1^), still raises concerns due to its bioaccumulative nature. Cd is known to accumulate in the kidneys and liver, and chronic exposure can lead to serious health problems, including renal failure and bone demineralization [35].

An important observation was that high soil concentrations [11] did not always correlate with high lettuce contamination levels, especially for As. This suggests that atmospheric deposition may play a significant role in crop contamination, especially in industrial areas such as Capuava. This highlights the complexity of pollution sources, including industrial emissions and urban air pollution, that influence the bioaccumulation of toxic elements in food.

### 2.2. Washing Procedure Effectiveness

**Figure 1:**
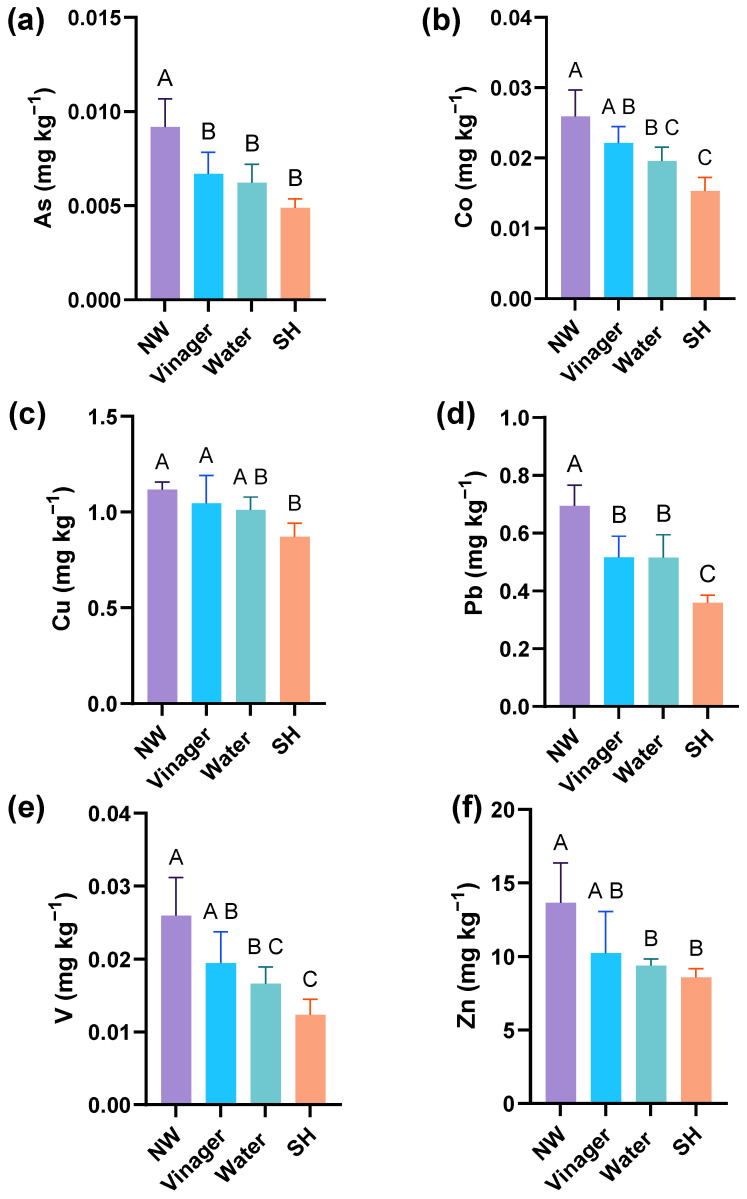
Concentration of (**a**) arsenic; (**b**) cobalt; (**c**) copper; (**d**) lead; (**e**) vanadium and (**f**) zinc in milligram per kilogram of lettuce fresh mass after the lettuces from Capuava 1 site were submitted to four different procedures: No-washing (NW); Vinegar immersion; Water immersion; Sodium Hypochlorite immersion (SH). Collums bar represents concentration means (n = 5). Different letters above the bars indicate differences in the same element among washing procedures according to Tukey’s test (*p* < 0.05). Whiskers correspond to the standard error for each bar.

Lettuce leaves are particularly susceptible to contaminant accumulation due to their large surface area and thin epidermis without a protective cuticle. In this study, different washing treatments—immersion in water, vinegar, and sodium hypochlorite (commercial bleach) were applied to lettuce samples from the Capuava 1 garden, which had the highest PTE concentrations. These methods reflect common household practices for washing vegetables and the results are summarized in Figure 1.

For elements such as Ba, Cd, Cr, and Ni, no significant differences were observed between the treatments, suggesting that washing does not effectively reduce these contaminants. Contrary to expectations, vinegar immersion did not outperform water in removing most contaminants. Although vinegar showed moderate removal efficiencies for As and Pb, it did not provide significant advantages over water. Specifically, vinegar removed 27% of As, 26% of Pb, and similar percentages for V, Zn, and Co, with minimal effect on Cu concentration.

Water immersion was more effective than vinegar for most elements, especially V, which was reduced by 36%, while As, Zn, and Pd were reduced by about 30%. However, water was less effective for elements that can penetrate deeper into plant tissues, such as Cd, Cr, and Ni.

Sodium hypochlorite (commercial bleach) showed the highest overall efficiency, particularly for Pb (48% reduction), As (46%), and V (52%). Its strong oxidizing properties make it highly effective at removing contaminants bound to organic matter on the leaf surface. Sodium hypochlorite also showed significant reductions for Co (41%) and Zn (37%), making it the most effective treatment for multiple contaminants.

Our results indicate that while washing with household chemicals can reduce the contaminant levels in urban garden vegetables, the effectiveness varies by contaminant and method. Sodium hypochlorite was particularly effective for several PTEs, suggesting that it may be a better option for reducing contaminant levels. However, the lack of significant differences between treatments for certain elements (Ba, Cd, Cr, Ni) suggests that washing alone may not be sufficient for some contaminants. These results suggest that is possible to reduce PTE concentrations in vegetables grown in urban gardens, but it is also crucial to consider broader strategies to reduce contamination at the source and improve overall soil quality.

### 2.3. Estimated Daily Intake

The EDIs of essential elements and PTEs from lettuce consumption were calculated and compared with reference values, as shown in Table 2. Considering all the urban gardens accessed in this study, the EDI was less than 1% of the limit value for the PTEs As, Ba, Ni, and V, indicating that lettuce is not a source of exposure to these elements. On the other hand, the calculated daily intake of Cd, Cu, and Zn represented from 1.6 to 4.4% of the reference values, while for Cr and Mo, the EDI can contribute with 5.7 to 8% of the recommended intake. It is worth mentioning that the EDI of lettuce grown in the urban gardens of Santo André can reach from 1.6 to 234% of the average intake of Pb.

Since Capuava 1 was the urban garden with the highest concentrations of chemical elements in this study, and it was possible to significantly reduce the levels of some of these elements by washing with sodium hypochlorite, the EDI was also calculated for lettuce from Capuava 1 unwashed and after washing. The results showed that for Cd, Cr, and Ni, the EDI before and after washing were very close, indicating that the washing process has little influence on their daily intake.

Washing was responsible for reducing the EDI for Cu and Ba from 15 to 22%. The EDIs for As, Co, Pb, V, and Zn were the most affected by the washing, showing a reduction of 37 to 53%. Even with this significant reduction, the EDI for Pb remains high, representing 6.8 to 1026% of the average intake of this element established by the Joint FAO/WHO Expert Committee on Food Additives (JECFA) [33].

The most common sources of Pb contamination in food crops are water and atmospheric deposition. According to JECFA [33], it has not been possible to establish a health-protective Provisional Tolerable Weekly Intake (PTWI) for Pb, considering its toxicology, epidemiology and exposure assessment. However, an average dietary exposure range of 0.02 to 3 µg kg^−1^ body weight day^−1^ was estimated [33], corresponding to 1.4 to 210 µg day^−1^, for a person of 70 kg body weight. An EDI closer to the lower end of this range (1.4 µg day^−1^) could be considered safe, as health risks at this level of exposure are negligible. On the other hand, an exposure level closer to 210 µg day^−1^ is of concern, because it was associated with a modest increase in the risk of cerebrovascular stroke and ischemic heart disease [33].

The EDI obtained for Pb in the worst-case scenario (unwashed Capuava 1) is 13% of the upper limit of the mean intake (210 µg day^−1^), and, therefore, even the daily consumption of this lettuce does not represent a high risk for the population of Santo André. However, it is necessary to balance the diet and be careful with other foods that may contain high levels of Pb. Finally, it is recommended to wash the lettuce with sodium hypochlorite, which can reduce the Pb EDI by 50%.

### 2.4. Risk Assessment

Table S1 shows the 95% upper confidence limit (UCL95) for the concentration of PTEs in lettuce samples from urban gardens in Santo André, along with the EDI, hazard quotient (HQ), and carcinogenic risk (CR) for adults and children. The data show the PTE concentrations before and after different washing treatments applied to lettuce from the Capuava 1 garden, which had the highest contamination levels among the gardens.

The HQ values indicate that the risk of Pb exposure was significantly higher for children than for adults, with a hazard quotient of 0.426 for children in unwashed lettuce from Capuava 1, compared to 0.139 for adults. After washing with sodium hypochlorite, the HQ for children was reduced to 0.198, showing a significant improvement. In terms of carcinogenic risk, children were more vulnerable than adults, especially for exposure to As and Pb. For example, the carcinogenic risk for children from Pb in unwashed lettuce from Capuava 1 was 1.27 × 10^−5^, which was reduced to 5.88 × 10^−6^ after washing with sodium hypochlorite, demonstrating the effectiveness of this washing method in reducing health risks. Other washing methods, such as vinegar and water, were less effective in reducing the concentrations of these contaminants, but still provide some risk reduction.

**Figure 2:**
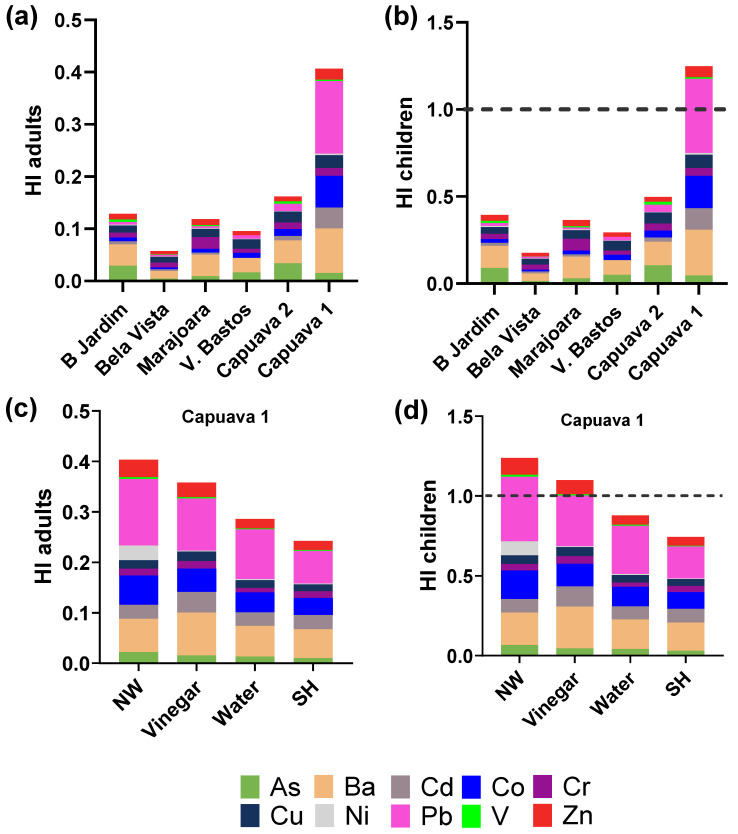
Hazard Index for (**a**) adults and (**b**) children from the consumption of lettuce from different urban gardens in Santo André. Hazard Index for (**c**) adults and (**d**) children from the consumption of lettuce from Capuava 1 site that were submitted to four different procedures: No-washing (NW); Vinegar immersion; Water immersion; Sodium Hypochlorite immersion (SH).

**Figure 3:**
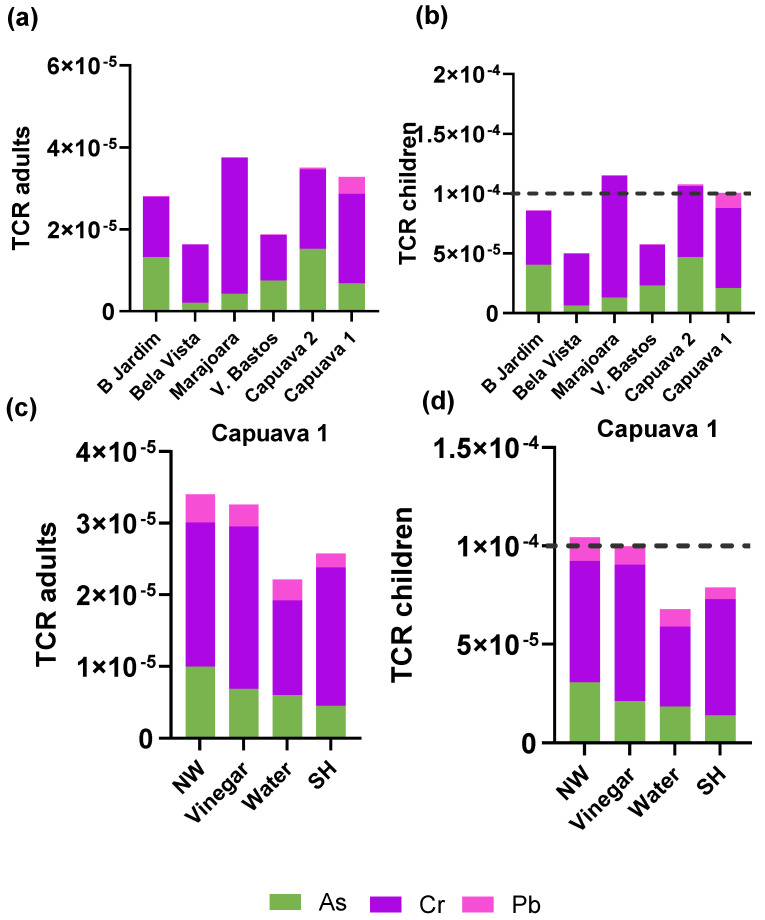
Total Cancer Risk for (**a**) adults and (**b**) children from the consumption of lettuce from different urban gardens in Santo André. Total Cancer Risk for (**c**) adults and (**d**) children from the consumption of lettuce from Capuava 1 site that were submitted to four different procedures: No-washing (NW); Vinegar immersion; Water immersion; Sodium Hypochlorite immersion (SH).

The results presented in Figure 2 and Figure 3 provide a comprehensive assessment of the non-cancer and cancer risks associated with the consumption of lettuce from urban gardens in Santo André, Brazil. Specifically, the analysis focuses on the Hazard Index (HI) and Total Cancer Risk (TCR) for both adults and children, highlighting the influence of different washing procedures on risk mitigation.

The HI assesses the non-cancer health risks associated with the ingestion of contaminants found in lettuce. An HI greater than 1 indicates a potential health concern. Figure 2a,b, show the HI values for adults and children consuming lettuce from six urban garden sites (Capuava 1, Capuava 2, Jardim, Bela Vista, Marajoara, and Vila Bastos). In adults, the HI remains consistently below 1 in all sites, indicating that the non-cancer risks of lettuce consumption are within acceptable limits. For children, however, the HI is significantly higher, approaching or exceeding the critical value of 1 in some sites, such as Capuava 1 and Marajoara. These elevated values suggest that children may be at a greater risk of non-cancer health effects from consuming lettuce grown in these locations.

The effects of different washing procedures: No-washing, Vinegar immersion, Water immersion, and Sodium Hypochlorite immersion on HI are shown in Figure 2c,d for lettuce from the Capuava 1 site. The results show a reduction in HI for both adults and children when washing procedures are applied, with sodium hypochlorite (SH) immersion being the most effective. This finding underscores the importance of implementing proper washing techniques to mitigate potential health risks associated with contaminant ingestion.

The Total Cancer Risk (TCR) evaluates the lifetime cancer risk from consuming contaminated lettuce, with a threshold of 1 × 10^−4^ considered as the upper limit of acceptability. Figure 3a,b show the TCRs values for adults and children consuming lettuce from the six urban gardens are presented. The TCR values for adults remained below the acceptable threshold in all studied gardens, indicating no significant cancer risk. However, for children, TCR values approach or exceed the acceptable limit in certain gardens (Marajoara, Capuava 1 and Capuava 2), particularly due to higher concentrations of Cr and Pb. This suggests that children are at a heightened risk of cancer from consuming contaminated lettuce, highlighting the need for more stringent monitoring and intervention measures.

The effect of washing procedures on the TCR for lettuce from Capuava 1 is detailed in Figure 3c,d. Similar to the HI results, the use of sodium hypochlorite immersion resulted in the most significant reduction in TCR for both adults and children. This suggests that effective washing techniques can significantly reduce cancer risk, further supporting the use of appropriate post-harvest treatments to reduce contaminant exposure.

In conclusion, the results highlight a remarkable spatial variation in health risks in the different urban gardens, with Capuava 1 and Capuava 2 being areas of particular concern for non-cancer risks and Marajoara, Capuava 1 and Capuava 2 for cancer risks, especially for children. In addition, the data demonstrate the critical role of washing procedures in reducing health risks, with sodium hypochlorite immersion proving to be the most effective method for r both non-cancer and cancer risks.

### 2.5. Lead Isotope Ratios

**Figure 4:**
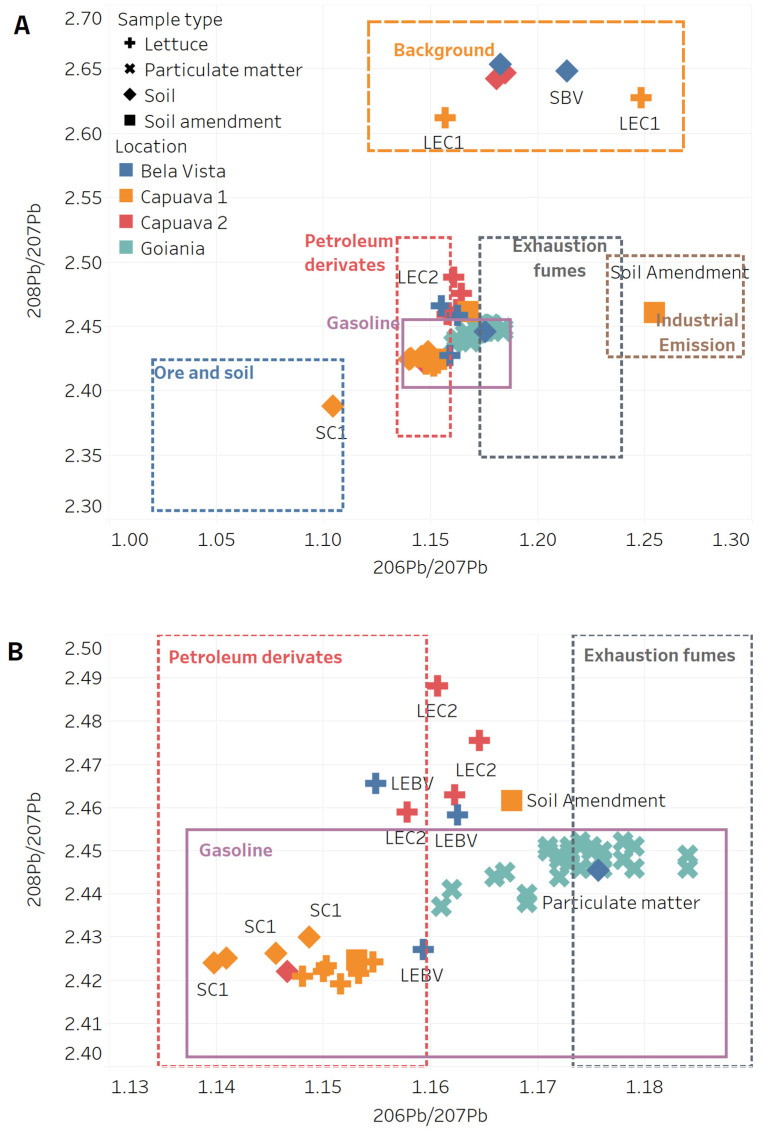
Isotopic ratios ^206^Pb/^207^Pb versus ^208^Pb/^207^Pb for soil, lettuce and soil amendment (this study) and particulate matter (**A**) [42] and details of Pb isotopic ratio usually observed to gasoline, petroleum derivatives and particulate matter (**B**). The boxes represent the ranges of isotopic ratio for ore and soil samples in China [28], Gasoline [40,42], Exhaustion fumes [42], petroleum and derivates [41] and background [40]. + Lettuce, × Particulate matter, ♦ soil, ■ soil amendment.

Figure 4 shows the Pb stable IRs of soils, lettuce from Capuava 1–2 and Bela Vista, and soil amendment from Capuava 1 in Santo André. These values were compared with the IR values of particulate matter from Goiania and other end members (pure chemical compounds) such as background [40], ores and soils [29], petroleum derivatives [41], gasoline [40,42], exhaust fumes and industrial emissions [42]. Considering these sources as potential end members, Capuava 1 soils with higher Pb concentrations had IR values compatible with Pb ores and soils but appeared to be partially influenced by petroleum and gasoline contributions. Two Bela Vista soils (designated SBV1 and 2) with low Pb concentration had IRs in the range of background values and SBV3 had an isotopic composition compatible with atmospheric Pb deposition from exhaust gases. This behavior is consistent with the closer location of Capuava to the Capuava petrochemical complex, while the Bela Vista soils were far from the direct influence of the petrochemical complex and had a similar IR background or particulate matter (Figure 4B) associated with vehicular traffic and emissions, as discussed in our previous study [11].

For the lettuce samples, the values of two washed Capuava samples, from which dust and surface soil deposits were removed were in the same isotopic range as the background soils. These results are in agreement with Hiller et al. [23] who reported that high ^206^Pb/^207^Pb IRs (>1.2) are associated with geogenic sources. By cleaning the leaves, the procedure helps to accurately determine the isotopic composition of Pb that has been absorbed by the leaves. This helps to identify the sources of Pb contamination [43]. All unwashed and two washed Capuava samples still showed a very narrow IR region compatible with the Pb isotopic signal of gasoline, indicating a contribution of atmospheric Pb deposition in this region (Figure 4B).

Bela vista samples showed a broader IR distribution, which may indicate a mixture of source signals from background soils, gasoline, and car exhaust contributing to affect the Pb IR. At Bela Vista, the atmospheric deposition over the leaves seems to largely affect the lettuce samples, once IR values are close to petroleum derivatives, particulate matter and exhaust fumes, clearly apart from the Bela Vista soil IRs. This indicates that the root–soil contamination route is not the largest issue affecting the lettuce samples in this area.

Capuava soil amendments have IRs associated with industrial emissions and in a zone in between the signal of particulate matter and petroleum derivatives, respectively, which could originate in the composting process and recycling of soil and other waste material and be affected by atmospheric Pb deposition.

## 3. Discussion

This study provides important insights into the contamination risks associated with urban agriculture, especially in environments affected by industrial activities and heavy vehicle emissions.

The concentrations of PTEs detected in the Santo André lettuce samples were significantly higher than those found in other Brazilian regions. For example, in Belo Horizonte [7], Cu ranged from 0.40 to 0.82 mg kg^−1^, Pb was undetected or measured up to 0.109 mg kg^−1^, and Cd ranged from 2 to 18 µg kg^−1^. In stark contrast, the results from Santo André showed Cu concentrations ranging from 0.63 to 1.73 mg kg^−1^, Pb concentrations ranging from 0.02 to 0.85 mg kg^−1^, and Cd concentrations ranging from 3 to 70 µg kg^−1^. This strong difference highlights the impact of industrial activities in this urban area and emphasizes the importance of localized studies to understand the specific sources of pollution affecting each region.

In addition, a comparative study conducted in peri-urban São Paulo [44] showed that Cd concentrations reached 0.9 mg kg^−1^, Pb levels were as high as 1.8 mg kg^−1^, and Zn levels were 88.5 mg kg^−1^, which are higher than those found in Santo André. However, Cu concentrations in peri-urban São Paulo were similar to those in Santo André, suggesting common pollution patterns, probably due to similar urban and industrial emissions. These results indicate the pervasive influence of industrialization on urban agriculture and reinforce the need for targeted pollution control efforts in both regions.

International comparisons further illustrate the varying degrees of contamination. A study conducted in Szeged, Hungary [45] found comparable Cu and Zn levels comparable to those found in Santo André, although concentrations of Cd, Pb, and Ni differed, reflecting different sources of contamination in the regions. In Hungary, Cu concentrations reached 10.22 mg kg^−1^ and Zn was 38.75 mg kg^−1^, values similar to those observed in Santo André (2.89 to 10.1 mg kg^−1^ for Zn). These similarities suggest that despite geographical and industrial differences, certain pollutants, such as Cu, may originate from common urban and industrial processes. However, the authors reported higher levels of certain contaminants, such as Pb and Ni, indicating the specific historical and environmental factors influencing contamination in Europe compared to Brazil.

For example, in New York City and Buffalo [46], Pb concentrations were significantly higher than in Santo André, with levels reaching up to 24 mg kg^−1^ due to legacy soil contamination from industrial activities and the widespread use of lead-based paints. Although Pb levels were lower in Santo André, they still represent a significant contamination risk, especially for vulnerable populations such as children, who may consume higher amounts of urban-grown vegetables.

A study conducted in Havana, Cuba [47] showed comparable contamination risks, with Pb and Cd levels posing significant health risks to urban gardeners and consumers. In Havana, as in Santo André, urban produce is an important food source, especially in economically disadvantaged communities. These comparisons with cities in both the Global North and Global South show that while patterns of contamination differ, urban agriculture is universally at risk from industrial pollutants, vehicular emissions, and historical soil contamination.

In evaluating the effectiveness of mitigation strategies, the study focused on washing methods. Sodium hypochlorite was found to be the most effective method for reducing surface contamination, reducing Pb, As, and Zn concentrations by up to 50%. However, the limited effectiveness of washing on other elements, such as Cd and Cr, suggests that deeper penetrating contaminants may not be easily removed by surface cleaning alone. Previous research by Egendorf et al. [19] in New York supports our findings, where various washing techniques reduced Pb contamination in lettuce by 75–94%, particularly from soil splashes. This reduction is higher than the 48% observed in Santo André, which may be due to differences in contamination sources and environmental conditions.

Interestingly, Egendorf et al. [19] found that vinegar soaks were more effective than water in removing Pb, while our study found the opposite trend. This discrepancy could be attributed to differences in the chemical composition of the soils and atmospheric pollutants in New York versus Santo André. Such findings reinforce the need for region-specific guidelines for mitigating PTE contamination in urban agriculture, as what works in one region may not be as effective in another.

In a study conducted in Vienna, Austria [48], the authors similarly evaluated washing techniques to assess their effectiveness in reducing PTE contamination in lettuce. Their results showed that while simple rinsing could effectively remove surface-bound elements such as V, Co, and Ni, it had little to no effect on elements such as Mo, Cd, Cu, Zn, and Ba. This suggests that certain elements are tightly bound within plant tissues rather than being surface contaminants, a finding consistent with our observations in Santo André.

The persistence of contaminants such as Cd and Cr in the Santo André samples even after washing underscores the need for comprehensive contamination mitigation strategies beyond surface cleaning. These findings support the use of more advanced methods, such as soil amendments or barriers, to prevent contaminants from reaching edible plant parts. The EDI reported in another study [35], conducted on lettuce plants grown in a greenhouse in São Bernardo do Campo (Brazil), a city of the “big ABC” region, showed the following values for Zn (89 µg day^−1^), Cd (0.24 µg day^−1^), Cu (21 µg day^−1^) and Cr (1.10 µg day^−1^) in the control group, which were lower than those obtained in the present study, indicating the influence of atmospheric pollution in open air urban gardens.

The bioaccumulation of heavy metals in urban-grown vegetables and the associated health risks have been the focus of several studies worldwide. For example, a study in Bologna, Italy [49] reported higher levels of Pb and Cd in urban-grown vegetables, that exceeded safe consumption limits for children. This is similar to the findings in Santo André, where children’s consumption of urban produce is a particular health concern due to their susceptibility to contaminants.

In addition, research in peri-urban China [50] showed that health risks, especially for children, were significant due to high levels of Cd, As, and Cr in vegetables, with HQ levels exceeding safe limits. This is consistent with the present study, where elevated HQ levels for children in the Capuava 1 and Marajoara gardens indicate an urgent need for public health interventions.

Lead isotope analysis proved to be invaluable in tracing the sources of contamination in the urban gardens of Santo André. Our results suggest that Pb atmospheric pollution, particularly from gasoline and exhaust fumes emissions, plays a significant role in a direct route of absorption by leaves in an area with soils that present natural Pb IRs. However, Pb atmospheric deposition also affects gardens in a broader and more ubiquitous manner, reaching lettuce samples through both soil and leaf absorption pathways A similar study in Vienna [48] used Pb isotope analysis to show that urban plants primarily absorbed Pb from soil, with atmospheric deposition playing a key role in contamination. The Vienna study, like ours, emphasizes the importance of distinguishing between natural and anthropogenic contamination sources.

Previous studies, including those by Egendorf et al. [19] and Trimmel et al. [48], support our findings on the partial effectiveness of washing methods and the value of Pb isotope analysis in identifying contamination sources. These results, together with the present study, suggest that atmospheric deposition plays a central role in contamination and that further interventions are needed to ensure the safety of urban agriculture.

To ensure the sustainability and safety of urban agriculture, comprehensive environmental management strategies must be implemented. These include reducing industrial emissions, improving soil remediation, and employing physical barriers such as mulch to prevent soil splashing [19], which is a known contributor to surface contamination. In addition, it is important to control the type of soil amendments used in urban gardens, as some amendments may inadvertently introduce or mobilize contaminants. Ensuring the quality of irrigation water is also critical, as contaminated water can be a significant source of heavy metals and other contaminants.

In addition, a key concern highlighted in this study is the increased vulnerability of children to contamination risks. Our findings reveal a distinct difference in non-cancer and cancer risks between adults and children. The Hazard Index (HI) values indicate that children face a significantly higher risk of non-cancer effects, particularly from Pb and Cr, in gardens such as Capuava and Marajoara. Similarly, the Total Cancer Risk (TCR) for children exceeds the threshold in these areas, underscoring the vulnerability of this population group to PTE exposure. Proper washing of vegetables has been shown to be an effective way to mitigate some of the risks, as it can significantly reduce surface contamination from pollutants such as Pb and Zn. However, the limited effectiveness of washing in removing deep-seated contaminants such as Cd and Cr highlights the need for more comprehensive solutions. The risk for children was calculated using the upper confidence limit of the 95th percentile (UCL95), which is an extremely conservative scenario to ensure that even the most vulnerable populations are also considered. Therefore, public health policies should not only encourage the consumption of fresh produce but also promote proper washing techniques as an important, though partial, mitigation strategy to reduce exposure to PTEs.

In summary, addressing contamination in urban agriculture requires a multifaceted approach, including improved environmental management, strict regulation of allowable contaminant levels, and targeted public health interventions. Future research should focus on developing more effective mitigation strategies and exploring innovative agricultural practices to reduce the risk of contamination and ensure long-term food safety and public health protection.

## 4. Materials and Methods

### 4.1. Sample Collection

**Figure 5:**
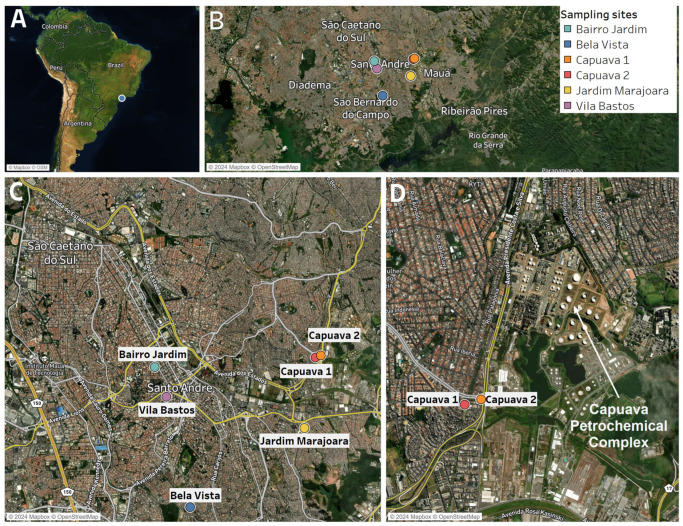
Study area with garden location (**A**,**B**) surrounded by main traffic streets and roads (**C**) and the proximity to Capuava Petrochemical Complex (**D**).

Lettuce samples were collected from urban gardens in the city of Santo André, a densely populated municipality within the MASP (Brazil). Known for its industrial history and rapid urbanization, Santo André has diverse urban horticultural sites with varying levels of exposure to environmental pollutants. These gardens, detailed in our previous study [11], are in different neighborhoods, providing a representative sample of the city’s complex urban landscape. For specific details on the sampling areas, including soil characteristics and proximity to potential sources of contamination, see Lange et al. [11]. The sites were identified as Capuava 1 (23°64′ S 46°49′ W), Capuava 2 (23°64′ S 46°48′ W), Jd Marajora (23°66′ S 46°49′ W), Bairro Jardim (23°65′ S 46°64′ W), Vila Bastos (23°66′ S 46°53′ W) and Bela Vista (23°69′ S 46°52′ W) (Figure 5).

The lettuce samples were collected following the guidelines of EMBRAPA [51], which recommends sampling at least one lettuce plant per 100 cultivated plants, using a knife commonly used by gardeners. A total of 64 lettuce plants. were collected. Many gardeners were reluctant to remove the roots of the collected plants to avoid damaging the beds. Therefore, only the concentration of trace elements in the lettuce leaves was analyzed. These leaves were then placed in plastic bags and sent immediately to the laboratory. The plant samples were washed under running water, and excess water was removed manually. The samples were then placed in paper bags and dried in an oven at 40 °C until a constant weight was reached.

### 4.2. Washing Test

Additional tests were conducted on lettuce samples from the plot with the highest concentration of trace elements (Capuava 1). These tests simulated domestic washing of the vegetable and evaluated the potential removal of trace elements by this process. For this purpose, five lettuce plants were collected, and the leaves of each plant were divided into four parts. The following treatments were applied: (i) no washing; after washing under running water: (ii) immersion in tap water for 20 min; (iii) immersion in 1 L of water containing one tablespoon of commercial vinegar (4% acidity) for 20 min; (iv) immersion with one litter of water containing one spoon of commercial sodium hypochlorite (2.5%) (commercial bleach) for 20 min. After the immersion period, the leaves were again washed under running water and dried at 40 °C. After the determination of trace element concentrations, a univariate ANOVA test (95% confidence level) was applied to the generated data to evaluate whether there were differences between the treatments.

### 4.3. Element Determination

The lettuce leaves were pre-cut with scissors. For the acid extraction of elements, approximately 200 mg of sample was pre-digested for 48 h in 2 mL of concentrated HNO_3_ (65% PA), which was previously sub-distilled. The samples were heated in a graphite digestion block at 95 °C for 4 h, according to the procedure described by Paniz et al. [52]. After cooling to room temperature, the samples were diluted to 40 mL with ultrapure water.

Elements were determined by inductively coupled plasma mass spectrometry (ICP-MS, Agilent 7900, Hachioji, Japan). The equipment was operated in a clean room using high-purity argon (99.999%, White Martins, Diadema, Brazil). A Mira MistTM nebulizer (Burgener Research Inc., Mississauga, ON, Canada) was used for sample introduction. The helium (He) (5 mL min^−1^ for He mode and 10 mL min^−1^ for HEHe—High Energy Helium mode) was used as the collision cell gas to minimize spectral interference for some elements. The instrumental conditions were the same as those described by Paniz et al. [52].

Stock solutions containing all elements were used (10 mg L^−1^) (Perkin Elmer, Norwalk, CT, USA), and plant reference materials (CRM 1573a—Tomato leaves certified by the National Institute of Standards and Technology; CRM Agro 1003A—Tomato leaves certified by EMBRAPA) were used for quality control of the determinations. The analytical results of the CRMs were in statistical agreement with the certified values.

### 4.4. Estimated Daily Intake of Chemical Elements from Lettuce

The estimated daily intake (EDI) of chemical elements from lettuce consumption was calculated for As, Ba, Cd, Co, Cr, Cu, Ni, Pb, V and Zn using the following Equation (1) [53]:

(1) $$EDI=DI_{vegetables\;intake}\times C_{element}$$

The EDI for each element, expressed as µg day^−1^, depends on the average daily intake of lettuce (DI), which was estimated to be 40 g day^−1^ FW, equivalent to about four leaves [54,55]. The mean element concentration in lettuce leaves (C_element_), expressed in µg g^−1^ FW, also influences the EDI.

In this study, the EDI was first calculated for the mean concentration obtained from all the urban gardens accessed, in order to obtain the mean intake of chemical elements through the consumption of lettuce from these urban gardens of Santo André. Due to the significant differences obtained for the concentrations of some elements in lettuce from Capuava 1 before and after washing with sodium hypochlorite, the EDI was also calculated in these two treatments (unwashed and washed with sodium hypochlorite), aiming to assess whether washing affected the intake of nutrients and PTEs.

### 4.5. Risk Assessment

The human health risk assessment was performed by evaluating both non-carcinogenic and carcinogenic risks for adults and children. The non-carcinogenic risk for each element was quantified using the hazard quotient (HQ), calculated as shown in Equation (2) [52,56,57]:

(2) HQ=EDI/RfD

where the EDI is the daily intake of each element in mg kg^−1^ day^−1^, taking into account the 95% upper confidence limit (UCL95) of the PTE concentration.

The reference dose (RfD) is an estimate of the level of daily exposure to a particular substance that is likely to be achieved without significant risk of adverse effects over a lifetime. The assessment is based on a body weight of 70 kg for adults and 22.8 kg for children. The RfD is typically expressed in milligrams of the substance per kilogram of body weight per day (mg kg^−1^ day^−1^) for As (0.0003), Ba (0.02), Cd (0.001), Co (0.0003), Cu (0.04), Cr (0.003), Ni (0.02), Pb (0.0035), V (0.005), and Zn (0.3) mg kg^−1^ day^−1^, respectively [58]. An HQ value greater than 1 indicates a potential non-carcinogenic risk, whereas an HQ value less than 1 indicates no significant non-carcinogenic hazard [11,55,57].

The HQ values for these 10 elements were then combined to estimate the Hazard Index (HI), which provides an estimate of the non-carcinogenic risk associated with long-term dietary exposure to these elements when considered together [57].

(3) $$HI=\sum_{i=1}^{n}HQ$$

An HI value greater than 1 suggests potential non-cancerous health effects, while an HI less than 1 indicates that chronic health risks are unlikely [58,59].

The carcinogenic risk (CR) for As, Cr, and Pb were estimated using Equation (4)

(4) CR=EDI×SF

(5) $$TCR=\sum_{i=1}^{n}CR$$

Here, EDI is the estimated daily intake of each carcinogenic (UCL95) PTE in mg^−^ kg^−1^ day^−1^, and SF is the slope factor for each element. The slope factors considered were 1.5 for As, 0.5 for Cr, and 0.0085 for Pb [58]. The total carcinogenic risk (TCR) is equal to the sum of the risk from all exposure pathways for all individual PTE (Equation (5)). The acceptable range of total risk for regulatory purposes is 10^−6^ to 10^−4^ [58,59]. For regulatory purposes, a TRC of 10^−4^ or greater indicates a potentially high risk, whether for a single element or multiple elements.

### 4.6. Lead Isotope Analysis

One of the capabilities of mass spectrometric techniques such as ICP-MS that has been widely explored in recent years is the determination of isotope ratios (IR) [60]. Geochemistry, food and forensic science, age dating, and metrology are some of the applications of IR [25,60].

In this study, Pb IRs were determined in lettuce, as well as in the soil of urban garden soils and soil amendments to potentially identify the Pb sources in two urban gardens, where the highest and lowest Pb concentrations were found. Lettuce samples were prepared as described in the “Element Determination’ section of this study and soil/soil amendments were prepared as described by Lange et al. [11]. Lead isotopic composition (^204^Pb, ^206^Pb, ^207^Pb, and ^208^Pb) was measured by ICP-MS (Agilent 7900, Hachioji, Japan). The signal obtained for the procedural Pb blanks was equivalent to less than 1% (soil) or 4% (lettuce) of the signal for the less concentrated samples. It was considered as negligible relative to Pb content in the lettuce and soil samples, and then blank corrections were not required for the isotopic data.

The Standard Reference Material NIST 981, which is the primary isotope reference material (iRM) for Pb IRs [60], was obtained from the National Institute of Standards and Technology (Common Lead Isotopic Standard, NIST, Gaithersburg, MD, USA). This iRM was analyzed repeatedly during the run to evaluate method accuracy and correct for instrument drift. The IRs obtained were: ^204^Pb/^206^Pb = 0.058895 ± 0.005823, ^207^Pb/^206^Pb = 0.91495 ± 0.01266, ^208^Pb/^206^Pb = 2.1680 ± 0.0090. The measured IRs were in good agreement with certified values (^204^Pb/^206^Pb = 0.059042 ± 0.000037, ^207^Pb/^206^Pb = 0.91464 ± 0.00033, ^208^Pb/^206^Pb = 2.1681 ± 0.0008).

The graphs representing isotopic ratios (Pb isotopic analysis) and other data visualizations were generated using Tableau Desktop (version 2023.2 Tableau Software, LLC, Seattle, WA, USA). The isotopic graph (Figure 4) was created by plotting the isotopic ratios (^206^Pb/^207^Pb versus ^208^Pb/^207^Pb) for lettuce and soil samples from this study to visually compare contamination other sources [29,40]. The ranges of isotopic ratio for ore and soil samples in China [28], Gasoline [40,42], Exhaustion fumes [42], petroleum and derivates [41] and background [40] were considered for comparison with lettuce, soil and soil amendment samples. The graphical analysis helped identify contributions from various contamination sources, including industrial emissions and atmospheric deposition.

### 4.7. Statistical Analysis

The data obtained in this study were analyzed using univariate analysis of variance (ANOVA) to evaluate the differences in contamination levels among the various washing treatments applied to lettuce samples. Tukey’s post hoc test was employed to perform multiple comparisons and identify statistically significant differences between the treatments (*p* < 0.05). All statistical analyses, as well as the generation of graphs for elemental concentrations and risk assessment, were conducted using GraphPad Prism version 10.2.3 (GraphPad Software, La Jolla, CA, USA). Results are expressed as means ± standard deviations (SD) for each treatment group, providing a clear representation of the variability within the data.

## 5. Conclusions

This study provides crucial insights into the contamination risks associated with urban-grown lettuce in a highly industrialized region. Elevated concentrations of potentially toxic elements (PTEs), particularly Pb, Cr, and As, were found in lettuce samples from gardens near industrial areas, posing significant health risks, especially to vulnerable populations like children. The Total Cancer Risk (TCR) for children exceeded the acceptable thresholds in some locations, underscoring the urgency of implementing targeted interventions and public health strategies.

The results demonstrate that common washing procedures, such as using sodium hypochlorite, can significantly reduce surface-bound contaminants like Pb, As, and V, but are less effective against deeper-penetrating elements such as Cd and Cr. Therefore, washing alone is insufficient to fully mitigate health risks. Broader environmental control measures, including industrial emission management, soil remediation, and the regulation of urban agricultural practices, are necessary to limit PTE accumulation in food crops.

Moreover, this study highlights the importance of health education in affected communities. Informing the public about contamination risks and promoting best practices, such as proper washing techniques and limiting consumption from high-risk areas, are vital steps in protecting public health. Policymakers and local authorities should prioritize risk management actions that include the continuous monitoring of PTEs in urban-grown produce and enforcing stricter regulations on pollution sources in industrial regions.

In conclusion, while urban agriculture remains a promising solution for food security, it requires a comprehensive approach to ensure the safety of the food produced. By addressing both the environmental sources of contamination and the mitigation techniques at the consumer level, this study lays the groundwork for future efforts to protect urban populations from the health risks associated with contaminated produce. Further research should explore long-term solutions, such as remediation and safer agricultural practices, to sustainably reduce PTE levels in urban environments.

## Figures and Tables

**Table 1:** Mean concentration, median, and standard deviation (SD) in mg kg^−1^ of fresh mass (FM) of potentially toxic elements in lettuce samples collected from urban gardens in Santo André. DL: detection limit and UCL95: 95% upper confidence limit (n = 64).

District		As	Ba	Cd	Co	Cr	Cu	Ni	Pb	V	Zn
Capuava 1	Mean	0.0074	2.72	0.0620	0.028	0.0659	1.63	0.078	0.77	0.0241	10.1
	Median	0.0073	2.67	0.0592	0.028	0.0622	1.68	0.076	0.81	0.0252	10.3
	SD	0.0004	0.19	0.0049	0.0028	0.0073	0.12	0.022	0.11	0.0024	0.8
Capuava 2	Mean	0.0087	0.981	0.0090	0.0048	0.0375	1.24	0.021	0.053	0.0190	4.35
	Median	0.0068	0.988	0.0083	0.0046	0.0353	1.27	0.022	0.057	0.0136	4.35
	SD	0.0044	0.260	0.0030	0.0017	0.0179	0.12	0.005	0.023	0.0130	0.25
Marajoara	Mean	0.0029	0.859	0.0047	0.0026	0.0630	0.905	0.023	0.0094	0.0116	4.63
	Median	0.0025	0.791	0.0043	0.0026	0.0574	0.879	0.017	0.0093	0.0094	4.58
	SD	0.0012	0.325	0.0021	0.0006	0.0399	0.139	0.015	0.0050	0.0061	0.71
B. Jardim	Mean	0.0054	1.13	0.0069	0.0025	0.0383	0.802	0.024	0.022	0.0148	3.40
	Median	0.0040	1.13	0.0065	0.0023	0.0415	0.800	0.020	0.020	0.0090	2.86
	SD	0.0042	0.16	0.0017	0.0006	0.0100	0.067	0.013	0.005	0.119	1.19
V. Bastos	Mean	0.0052	0.840	<DL	0.0045	0.0295	1.13	0.030	0.028	<DL	3.70
	Median	0.0047	0.841	<DL	0.0043	0.0285	1.13	0.030	0.027	<DL	3.67
	SD	0.0018	0.074	<DL	0.0006	0.0051	0.12	0.002	0.006	<DL	0.36
Bela Vista	Mean	0.0020	0.459	0.0059	0.0015	0.0385	0.709	0.028	0.016	0.0057	2.89
	Median	0.0020	0.466	0.0063	0.0015	0.0410	0.746	0.028	0.015	0.0058	2.81
	SD	0.0004	0.046	0.0008	0.0002	0.0125	0.066	0.006	0.003	0.0012	0.35
Total	Mean	0.0050	1.06	0.0110	0.0051	0.0495	0.102	0.028	0.082	0.0146	4.61
	Median	0.0038	0.94	0.0061	0.0028	0.0426	0.955	0.022	0.020	0.0099	4.35
	SD	0.0036	0.57	0.161	0.0068	0.0301	0.269	0.019	0.206	0.0098	1.86
	UCL95	0.0125	2.66	0.0591	0.0270	0.0865	1.56	0.076	0.777	0.0377	10.1
Decree Law 55871 of 1965 [32]		1.00		1.00		0.10	30.0	5.00	0.50		50.0
RDC Nº 42 [31]		0.30		0.20					0.30		
Codex Alimentarius [33]				0.20					0.30		

**Table 2:** Estimated Daily Intake (EDI) of chemical elements through the consumption of lettuce from all urban gardens, as well as from lettuce of Capuava 1 urban garden unwashed and washed with sodium hypochlorite, and reference intake for each element.

Estimated Daily Intake				
Mean Intake (µg day^−1^) (% of the Reference Intake)				
	All Samples	Capuava 1 Unwashed	Capuava 1 Washed	Reference Intake
As	0.20 (0.04–0.95)	0.37 (0.07–1.7)	0.20 (0.03–0.93)	21–560 ^a^
Ba	44 (0.31)	80 (0.57)	68 (0.49)	14,000 ^b^
Cd	0.40 (1.6)	1.68 (6.7)	1.60 (6.4)	25 ^c^
Co	0.20	1.04	0.60	
Cr	2.00 (5.7–8.0)	2.32 (6.6–9.3)	2.32 (6.6–9.3)	25 W–35 M ^d^
Cu	40 (4.4)	45 (5.0)	35 (3.9)	900 ^e^
Ni	1.12 (0.11)	1.84 (0.18)	1.68 (0.17)	1000 ^f^
Pb	3.28 (1.6–234)	28 (13–1980)	14 (6.8–1026)	1.4–210 ^g^
V	0.60 (0.03)	1.04 (0.06)	0.48 (0.03)	1800 ^f^
Zn	184 (1.7–2.3)	548 (5.0–6.8)	344 (3.1–4.3)	8000 W–11,000 M ^e^

^a^ Benchmark dose lower confidence limit (BMDL) [36]. ^b^ Tolerable Daily Intake (TDI) [37]. ^c^ Tolerable Daily Intake (TDI) calculated from Tolerable Weekly Intake (TWI)/7 [33]. ^d^ Adequate Intake (AI) [38]. ^e^ Recommended Dietary Allowance (RDA) [38,39]. ^f^ Tolerable upper intake levels (UL) [38]. ^g^ Mean Intake [33]. Considering a 70 kg body weight person.

## Data Availability

Data sharing is not applicable to the article as datasets were generated or analyzed during the current study.

## Supplementary Materials

The following supporting information can be downloaded at: https://www.mdpi.com/article/10.3390/plants13192807/s1, Table S1: 95% upper confidence limit (UCL95) concentration of each PTE in urban gardens of Santo André and after washing procedures in lettuces from Capuava 1; Estimated Daily Intake (EDI); Hazard Quotient (HQ) and Carcinogenic Risk (CR) considering adults and children.
