# Supplementary material for: Potentially Toxic Elements in Urban-Grown Lettuce: Effectiveness of Washing Procedures, Risk Assessment, and Isotopic Fingerprint
Source: Plants (Basel). 2024 Oct 7;13(19):2807. doi: 10.3390/plants13192807 (PMC11479218; doi:10.3390/plants13192807)
Supplement: Supplementary file 1 [file plants-13-02807-s001.zip › plants-3237873-supplementary.pdf]

Supplementary Material

Potentially Toxic Elements in Urban-Grown Lettuce: Effectiveness of Washing Procedures, Risk Assessment, and Isotopic Fingerprint

Camila Neves Lange[1,2*], Bruna Moreira Freire[1], Lucilena Rebelo Monteiro[2], Marycel Elena Barboza Cotrim[2], Bruno Lemos Batista[1*]

[1] *Federal University of ABC (UFABC), Center for Natural and Human Sciences (CCNH), Santo André, São Paulo, Brazil, 09210-580*

[2] *Instituto de Pesquisas Energéticas e Nucleares IPEN-CNEN, São Paulo, SP, Brazil, 05508-000.*

Table S1. 95% upper confidence limit (UCL95) concentration of each PTE in urban gardens of Santo André and after washing procedures in lettuces from Capuava 1; Estimated Daily Intake (EDI); Hazard Quotient (HQ) and Carcinogenic Risk (CR) considering adults and children.

	B Jardim	Bela Vista	Marajoara	Vila Bastos	Capuava 2	Capuava 1	Capuava 1 – Washing Procedures			
							No-washing	Sodium Hipochloride	Vinager	Water
UCL95 μg kg^{-1}										
As	0.015	0.002	0.005	0.009	0.018	0.008	0.012	0.005	0.008	0.007
Ba	1.44	0.501	1.43	0.950	1.54	2.99	2.31	2.01	2.97	2.12
Cd	0.010	0.006	0.007	<LQ	0.014	0.070	0.048	0.050	0.072	0.047
Co	0.004	0.002	0.004	0.005	0.007	0.032	0.031	0.018	0.024	0.021
Cr	0.051	0.050	0.116	0.039	0.068	0.076	0.070	0.067	0.079	0.046
Cu	0.908	0.749	1.12	1.28	1.47	1.73	1.19	0.986	1.29	1.08
Ni	0.064	0.034	0.062	0.032	0.028	0.110	0.991	0.046	0.061	0.072
Pb	0.032	0.019	0.019	0.040	0.084	0.850	0.810	0.394	0.634	0.600
V	0.040	0.007	0.023	<LQ	0.045	0.026	0.033	0.015	0.026	0.019
Zn	5.80	3.28	5.89	4.29	4.84	10.87	18.25	9.47	15.04	9.90
EDI adults (UCL95) (mg/kg/day)										
As	8.77E-06	1.35E-06	2.83E-06	4.99E-06	1.02E-05	4.56E-06	6.65E-06	3.02E-06	4.60E-06	4.02E-06
Ba	8.22E-04	2.86E-04	8.20E-04	5.43E-04	8.80E-04	1.71E-03	1.32E-03	1.15E-03	1.70E-03	1.21E-03
Cd	5.62E-06	3.67E-06	3.83E-06	<LQ	8.15E-06	4.02E-05	2.76E-05	2.83E-05	4.12E-05	2.68E-05
Co	2.14E-06	1.02E-06	2.21E-06	3.11E-06	3.96E-06	1.83E-05	1.75E-05	1.02E-05	1.38E-05	1.19E-05
Cr	2.93E-05	2.83E-05	6.64E-05	2.21E-05	3.88E-05	4.36E-05	4.02E-05	3.85E-05	4.51E-05	2.63E-05
Cu	5.19E-04	4.28E-04	6.39E-04	7.33E-04	8.39E-04	9.90E-04	6.79E-04	5.63E-04	7.36E-04	6.19E-04
Ni	3.64E-05	1.92E-05	3.56E-05	1.86E-05	1.60E-05	6.31E-05	5.66E-04	2.65E-05	3.49E-05	4.10E-05
Pb	1.85E-05	1.08E-05	1.11E-05	2.29E-05	4.81E-05	4.86E-04	4.63E-04	2.25E-04	3.62E-04	3.43E-04
V	2.30E-05	3.86E-06	1.29E-05	<LQ	2.58E-05	1.49E-05	1.87E-05	8.52E-06	1.47E-05	1.09E-05
Zn	3.31E-03	1.87E-03	3.37E-03	2.45E-03	2.77E-03	6.21E-03	1.04E-02	5.41E-03	8.59E-03	5.66E-03
EDI children (UCL95) (mg/kg/day)										
As	2.69E-05	4.14E-06	8.69E-06	1.53E-05	3.12E-05	1.40E-05	2.04E-05	9.26E-06	1.41E-05	1.24E-05
Ba	2.52E-03	8.79E-04	2.52E-03	1.67E-03	2.70E-03	5.24E-03	4.05E-03	3.52E-03	5.21E-03	3.72E-03
Cd	1.73E-05	1.13E-05	1.17E-05	<LQ	2.50E-05	1.23E-04	8.48E-05	8.69E-05	1.27E-04	8.23E-05
Co	6.58E-06	3.14E-06	6.79E-06	9.54E-06	1.22E-05	5.60E-05	5.37E-05	3.13E-05	4.24E-05	3.66E-05
Cr	9.01E-05	8.69E-05	2.04E-04	6.80E-05	1.19E-04	1.34E-04	1.23E-04	1.18E-04	1.38E-04	8.09E-05
Cu	1.59E-03	1.31E-03	1.96E-03	2.25E-03	2.57E-03	3.04E-03	2.08E-03	1.73E-03	2.26E-03	1.90E-03
Ni	1.12E-04	5.91E-05	1.09E-04	5.70E-05	4.90E-05	1.94E-04	1.74E-03	8.13E-05	1.07E-04	1.26E-04
Pb	5.67E-05	3.33E-05	3.40E-05	7.03E-05	1.48E-04	1.49E-03	1.42E-03	6.92E-04	1.11E-03	1.05E-03
V	7.07E-05	1.19E-05	3.95E-05	<LQ	7.93E-05	4.59E-05	5.76E-05	2.61E-05	4.52E-05	3.35E-05
Zn	1.02E-02	5.75E-03	1.03E-02	7.53E-03	8.50E-03	1.91E-02	3.20E-02	1.66E-02	2.64E-02	1.74E-02

Table S1. CONTINUTATION

	B Jardim	Bela Vista	Marajoara	Vila Bastos	Capuava 2	Capuava 1	Capuava 1 – Washing Procedures			
							No-washing	Sodium Hipochloride	Vinager	Water
HQ adults										
As	2.92E-02	4.49E-03	9.44E-03	1.66E-02	3.38E-02	1.52E-02	2.22E-02	1.01E-02	1.53E-02	1.34E-02
Ba	4.11E-02	1.43E-02	4.10E-02	2.71E-02	4.40E-02	8.54E-02	6.59E-02	5.74E-02	8.48E-02	6.06E-02
Cd	5.62E-03	3.67E-03	3.83E-03	<LQ	8.15E-03	4.02E-02	2.76E-02	2.83E-02	4.12E-02	2.68E-02
Co	7.14E-03	3.41E-03	7.38E-03	1.04E-02	1.32E-02	6.08E-02	5.83E-02	3.39E-02	4.61E-02	3.97E-02
Cr	9.78E-03	9.44E-03	2.21E-02	7.38E-03	1.29E-02	1.45E-02	1.34E-02	1.28E-02	1.50E-02	8.78E-03
Cu	1.30E-02	1.07E-02	1.60E-02	1.83E-02	2.10E-02	2.48E-02	1.70E-02	1.41E-02	1.84E-02	1.55E-02
Ni	1.82E-03	9.62E-04	1.78E-03	9.28E-04	7.99E-04	3.16E-03	2.83E-02	1.32E-03	1.74E-03	2.05E-03
Pb	5.28E-03	3.10E-03	3.17E-03	6.54E-03	1.38E-02	1.39E-01	1.32E-01	6.44E-02	1.04E-01	9.80E-02
V	4.61E-03	7.73E-04	2.58E-03	<LQ	5.17E-03	2.99E-03	3.75E-03	1.70E-03	2.95E-03	2.18E-03
Zn	1.10E-02	6.25E-03	1.12E-02	8.17E-03	9.23E-03	2.07E-02	3.48E-02	1.80E-02	2.86E-02	1.89E-02
HQ children										
As	8.97E-02	1.38E-02	2.90E-02	5.10E-02	1.04E-01	4.67E-02	6.80E-02	3.09E-02	4.71E-02	4.12E-02
Ba	1.26E-01	4.40E-02	1.26E-01	8.34E-02	1.35E-01	2.62E-01	2.02E-01	1.76E-01	2.60E-01	1.86E-01
Cd	1.73E-02	1.13E-02	1.17E-02	<LQ	2.50E-02	1.23E-01	8.48E-02	8.69E-02	1.27E-01	8.23E-02
Co	2.19E-02	1.05E-02	2.26E-02	3.18E-02	4.05E-02	1.87E-01	1.79E-01	1.04E-01	1.41E-01	1.22E-01
Cr	3.00E-02	2.90E-02	6.79E-02	2.27E-02	3.97E-02	4.46E-02	4.11E-02	3.94E-02	4.62E-02	2.70E-02
Cu	3.98E-02	3.29E-02	4.90E-02	5.63E-02	6.44E-02	7.60E-02	5.21E-02	4.32E-02	5.65E-02	4.75E-02
Ni	5.59E-03	2.95E-03	5.47E-03	2.85E-03	2.45E-03	9.69E-03	8.69E-02	4.06E-03	5.35E-03	6.29E-03
Pb	1.62E-02	9.51E-03	9.72E-03	2.01E-02	4.22E-02	4.26E-01	4.06E-01	1.98E-01	3.18E-01	3.01E-01
V	1.41E-02	2.37E-03	7.91E-03	<LQ	1.59E-02	9.18E-03	1.15E-02	5.23E-03	9.05E-03	6.69E-03
Zn	3.39E-02	1.92E-02	3.45E-02	2.51E-02	2.83E-02	6.36E-02	1.07E-01	5.54E-02	8.79E-02	5.79E-02
CR adults										
As	1.32E-05	2.02E-06	4.25E-06	7.48E-06	1.52E-05	6.84E-06	9.97E-06	4.53E-06	6.9E-06	6.03E-06
Cr	1.47E-05	1.42E-05	3.32E-05	1.11E-05	1.94E-05	2.18E-05	2.01E-05	1.93E-05	2.26E-05	1.32E-05
Pb	1.57E-07	9.21E-08	9.42E-08	1.95E-07	4.09E-07	4.13E-06	3.94E-06	1.92E-06	3.08E-06	2.91E-06
CR children										
As	4.04E-05	6.20E-06	1.30E-05	2.30E-05	4.68E-05	2.10E-05	3.06E-05	1.39E-05	2.12E-05	1.85E-05
Cr	4.50E-05	4.35E-05	1.02E-04	3.40E-05	5.95E-05	6.69E-05	6.17E-05	5.91E-05	6.92E-05	4.04E-05
Pb	4.82E-07	2.83E-07	2.89E-07	5.98E-07	1.26E-06	1.27E-05	1.21E-05	5.88E-06	9.45E-06	8.95E-06
